# The role of autophagic kinases in regulation of axonal function

**DOI:** 10.3389/fncel.2022.996593

**Published:** 2022-09-26

**Authors:** Sarah H. Berth, Dominick J. Rich, Thomas E. Lloyd

**Affiliations:** Department of Neurology, School of Medicine, Johns Hopkins University, Baltimore, MD, United States

**Keywords:** autophagy, kinase, axon, ULK1, mTOR, TBK1, ALS

## Abstract

Autophagy is an essential process for maintaining cellular homeostasis. Highlighting the importance of proper functioning of autophagy in neurons, disruption of autophagy is a common finding in neurodegenerative diseases. In recent years, evidence has emerged for the role of autophagy in regulating critical axonal functions. In this review, we discuss kinase regulation of autophagy in neurons, and provide an overview of how autophagic kinases regulate axonal processes, including axonal transport and axonal degeneration and regeneration. We also examine mechanisms for disruption of this process leading to neurodegeneration, focusing on the role of TBK1 in pathogenesis of Amyotrophic Lateral Sclerosis.

## Introduction

Autophagy is a cellular homeostatic process in which proteins, organelles, and cellular debris are sequestered, packaged, and delivered to the lysosome for degradation (Maday, 2016; Malik et al., 2019). This recycling process is essential for cell growth, survival, and development, as it regenerates raw materials including carbohydrates, lipids, and proteins, for use in a variety of metabolic processes (Sridharan et al., 2011; Xiang et al., 2020).

**FIGURE 1:**
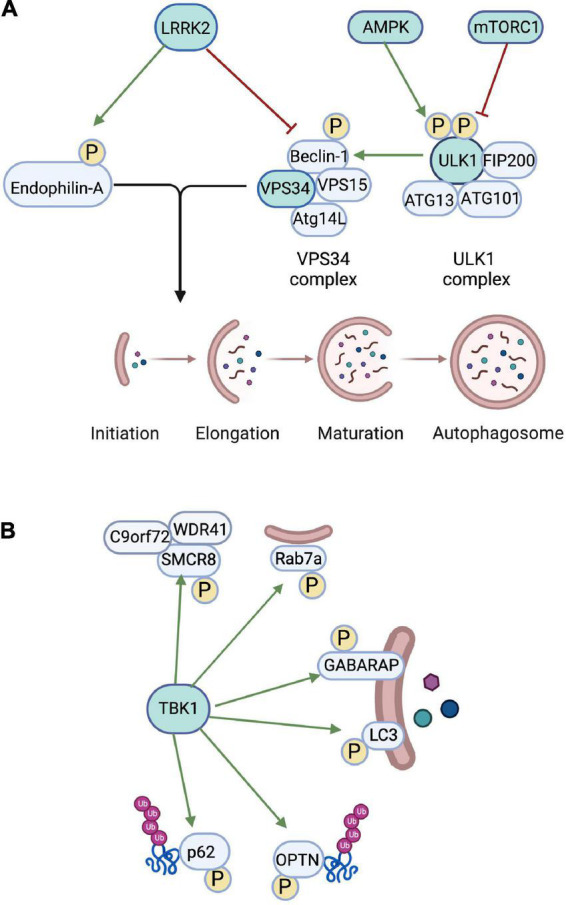
Role of autophagic kinases in regulating autophagy. **(A)** The autophagic kinase AMPK phosphorylates ULK1 to activate autophagy, while mTORC1 phosphorylates ULK1 to inhibit autophagy. ULK1 then phosphorylates Beclin-1 in the VPS34 complex to activate VPS34. VPS34 phosphorylates phosphatidylinositol to regulate the growing autophagosome membrane. The kinase LRRK2 phosphorylates Endophilin-A, which promotes the formation of autophagosome membranes. LRRK2 also phosphorylates Beclin-1 to inhibit the VSP34 complex. **(B)** TBK1 regulates initiation of autophagy *via* phosphorylation of several autophagic proteins. TBK1 phosphorylates SMCR8, which exists in a complex with WDR41 and C9orf72 and regulates autophagic flux. TBK1 phosphorylation of Rab7A targets damaged mitochondria to autophagosomal membranes. TBK1 also directly phosphorylates GABARAP and LC3, which prevents premature removal of GABARAP and LC3 from autophagosomal membranes. TBK1 enhances the targeting of ubiquinated proteins to the phagophore *via* phosphorylation of the adaptor proteins OPTN and p62. Created in BioRender.com.

Autophagy occurs in three distinct forms: chaperone mediated autophagy (CMA), microautophagy, and macroautophagy. Macroautophagy is the most well-studied of the three mechanistically distinct forms of autophagy, and its role in neurodegeneration has been widely explored. Macroautophagy is characterized by the formation of a double-membrane structure surrounding cytosolic cargoes (Figure 1A), and the maturation and transport of the autophagosome to the lysosome for degradation of its internal components (Glick et al., 2010; Wang et al., 2018). Macroautophagy can be further subdivided into organelle-specific processes including endoplasmic reticulum (ER-phagy), mitochondria (mitophagy), lysosomes (lysophagy) and nuclei (nucleophagy) (Heo et al., 2015; Malik et al., 2019). This review will focus on macroautophagy (hereafter referred to as autophagy) as the primary process involved in maintenance of cellular homeostasis in neuronal populations (Fleming et al., 2022).

Neurons, unlike most cell types, are post-mitotic and thus cannot dilute toxic material through cell division. Therefore, autophagy is particularly important for survival of neurons that must last the organism’s lifetime, and autophagy has been observed to occur constitutively in neurons (Maday et al., 2012; Maday and Holzbaur, 2014). This baseline level of autophagy occurs in a highly compartmentalized manner, with autophagosome biogenesis in the distal axon near synapses, maturation as the autophagosome is transported retrogradely toward the soma, and eventual fusion with the lysosome and degradation of its contents occurring at or near the cell body (Yue, 2007; Maday and Holzbaur, 2016). Potential sources of membrane for autophagosome biogenesis include endoplasmic reticulum (ER) (Hayashi-Nishino et al., 2009; Yla-Anttila et al., 2009; Hamasaki et al., 2013; Zhao et al., 2018), mitochondria (Hailey et al., 2010) and plasma membrane (Ravikumar et al., 2010; Nascimbeni et al., 2017). Of these, ER is the likely source of membranes in distal axons of neurons, as autophagosome formation has been observed at DFCP1-positive ER subdomains in the distal axon (Maday and Holzbaur, 2014). Axonal autophagy and synaptic function are highly interlinked. For example, Endophilin-A and Synaptojanin, proteins important for synaptic vesicle endocytosis, can regulate autophagy (George et al., 2016; Soukup et al., 2016; Vanhauwaert et al., 2017), while the presynaptic scaffolding protein Bassoon inhibits autophagic biogenesis (Okerlund et al., 2017). Synaptic activity regulates autophagy *via* modification of the presynaptic location of the core autophagy protein Atg9 (Yang et al., 2022). On the other hand, autophagy regulates synaptic activity, as impaired autophagy causes axonal ER accumulation and increased neurotransmission (Kuijpers et al., 2021). During autophagosome maturation and retrograde transport along the axon, autophagosomes switch from bidirectional to primarily retrograde movement along axons, develop increased amounts of cathepsin and undergo acidification (Katsumata et al., 2010; Maday et al., 2012). Almost all degradation occurs within mature lysosomes near the soma (Maday and Holzbaur, 2016; Cheng et al., 2018). In rodent models, it is long established that suppression of this neuronal autophagy is sufficient to induce abnormal protein aggregation and eventual neurodegeneration, underscoring the important role of autophagy in neuronal homeostasis and survival (Hara et al., 2006; Komatsu et al., 2006).

## Autophagic kinase involvement

In many cell types, autophagy is a tightly regulated degradative mechanism utilized primarily during periods of cellular starvation (Kim et al., 2002; Komatsu et al., 2005). Autophagic signaling and initiation require precise coordination of several autophagic kinases and adaptor molecules capable of sensing such changes in a cellular environment. In nutrient-rich conditions, autophagy is normally inhibited. However, in periods of cellular starvation, autophagy is activated to begin recycling of intracellular materials for metabolic availability. This nutrient-dependent regulation is facilitated by two critical kinases that act as master sensors for autophagy: the mammalian target of Rapamycin (mTOR) and adenosine monophosphate activated protein kinase (AMPK). Under nutrient rich conditions, mTOR complexes with Raptor and mammalian lethal with Sec13 protein 8 (mLST8) to form the mTOR Complex 1 (mTORC1) (Hara et al., 2002; Kim et al., 2002). mTORC1 acts as a master sensor for autophagy initiation, most notably through inhibition of Unc-51-like Kinase 1 (ULK1) *via* phosphorylation (Figure 1A). ULK1, the mammalian ortholog of yeast Atg1, is a Serine/Threonine Kinase that plays a critical role in autophagy initiation by forming complexes with FIP200, ATG13, and ATG101 (Chen et al., 2014; Xiang et al., 2020). mTORC1 binds to this ULK1 complex to phosphorylate ULK1 Ser 757, inhibiting this complex and preventing ULK1-induced autophagosome biogenesis (Kim et al., 2011; Saxton and Sabatini, 2017). TORC1 can phosphorylate multiple Ser sites on Atg13, inhibiting its ability to bind to ULK1 and form the ULK1 complex (Kamada et al., 2010). Conversely, when the cell lacks amino acid substrates and other necessary nutrients, inactivation of mTORC1 allows dephosphorylation and activation of the ULK1 complex. The ULK1 complex then localizes to the isolation membrane where its kinase activity initiates formation of the early phagophore (Ganley et al., 2009; Hosokawa et al., 2009).

AMPK plays an opposite but similarly critical role in sensing cellular metabolism and energy levels to tightly regulate autophagy initiation. In low energy conditions where detectable levels of cellular cyclic adenosine monophosphate (cAMP) drop, AMPK promotes autophagy by directly phosphorylating ULK1 (Figure 1A) at its Serine 313 and Serine 777 phosphorylation sites (Kim et al., 2011). This phosphorylation promotes formation of the ULK1 complex and initiates autophagy cascades (Tong et al., 2020).

The activated ULK1 complex has a number of downstream targets, some of which enable subsequent phagophore formation. ULK1 directly phosphorylates the downstream VPS34 complex to enable phagophore formation (Figure 1A). Vacuolar protein sorting 34 (VPS34) is the sole mammalian class III phosphoinositide 3-kinase (PI3K) critical for lipidation of the newly forming phagophore. VPS34 lipid kinase phosphorylates phosphatidylinositol to produce phosphatidylinositol 3-phosphate, a constituent of the autophagosome membrane (Kihara et al., 2001; Obara et al., 2006). *In vitro* studies have shown that either nutrient deprivation or inhibition of mTORC1 activity by Torin-1, an mTOR catalytic inhibitor, are sufficient to inhibit ULK1 Ser757 site phosphorylation and increase downstream phosphorylation of Beclin-1 Ser14, indicating that disinhibition of ULK1 allows phosphorylation of its downstream targets (Russell et al., 2013). Further, Atg14L was observed to bind to Beclin-1 and increase phosphorylation by ULK1, indicating that Atg14L is also a critical component of this complex (Russell et al., 2013). Altogether, this indicates that during autophagy induction, ULK1 phosphorylates downstream Atg14L-bound Beclin-1, which complexes with VPS34 to form the PI3K III complex, which can then be localized to the growing phagophore to phosphorylate phosphatidylinositol to produce phosphatidylinositol 3-phosphate for the initial autophagosome membrane (Figure 1A).

Studies of autophagosome biogenesis in *Drosophila* motor neurons have shown that Endophilin-A, known to be required for endocytosis at synapses, can act as a regulator of autophagy by promoting the formation of curved membranes and recruiting autophagy machinery and adaptor proteins to the newly formed phagophore (Soukup et al., 2016). Leucine-rich repeat kinase 2 (LRRK2) regulates phosphorylation of Endophilin-A (Figure 1A) at the Ser58 site, thus controlling phagophore membrane formation and regulating autophagy activation. Additionally, LRRK2 phosphorylates the Serine 295 phosphorylation site on Beclin-1 (Figure 1A), inhibiting Beclin-1 (and thus the VPS34 complex), further supporting the role of LRRK2 as an important inhibitory regulator of autophagy initiation (Manzoni et al., 2018; Takagawa et al., 2018).

Tank Binding Kinase 1 (TBK1) is a Serine/Threonine Kinase in the IKK Kinase family. TBK1 regulates diverse cellular processes including oncogenesis, neuroinflammation, lipid metabolism, and autophagy. TBK1 plays a major role in autophagy and mitophagy, specifically through phosphorylation of autophagy adaptor proteins for efficient cargo recruitment to the nascent autophagosome. TBK1 activation occurs *via* a multistep process involving K63-linked polyubiquitination of the Lys30 and Lys401 residues of TBK1, followed by phosphorylation of Ser373, inducing a conformational change in the Ser/Thr Kinase Domain (Tu et al., 2013; Oakes et al., 2017). Activated TBK1 acts as a positive regulator of autophagic adaptor proteins (Figure 1), including Sequestosome 1 (p62/SQSTM1) and Optineurin (OPTN). Activated TBK1 can phosphorylate Ser403 on the autophagy adaptor protein p62/SQSTM1, coordinating its recruitment to the autophagic machinery and initiating its role in autophagic clearance and recruitment of OPTN to mitochondria to initiate mitophagy (Pilli et al., 2012; Matsumoto et al., 2015). TBK1 can also directly phosphorylate Ser72 on RAB7A (Figure 1B), a late endosome protein that is recruited to depolarized mitochondria to promote mitophagy through the PINK1-Parkin pathway (Heo et al., 2018). TBK1 can also affect autophagy *via* regulation of the ULK1 complex (Vargas et al., 2019) or *via* direct phosphorylation of the autophagosome membrane components LC3 and GABARAP-L2 (Herhaus et al., 2020) (Figure 1B). Finally, TBK1 phosphorylates SMCR8, which exists in a complex with WDR41 and C9orf72, to regulate autophagic flux (Sellier et al., 2016; Sullivan et al., 2016). As described below, recent studies have implicated TBK1 in neurodegeneration, though the precise mechanisms remain unclear (Figure 1B).

## Regulation of axonal function by autophagic kinases

### Autophagy biogenesis and axonal transport in neurons

Axonal transport (AT) is a highly regulated process that utilizes the kinesin and dynein ATPase motor proteins to deliver organelles along microtubule tracks. Growing evidence suggests that autophagosomal maturation and AT are linked (Maday, 2016). AT is highly regulated by phosphotransferases (Brady and Morfini, 2017), and in fact, several autophagic kinases regulate AT. For example, in *Drosophila*, the ortholog of ULK1 (*atg1*) regulates anterograde synaptic vesicle AT through phosphorylation of the kinesin heavy chain adaptor UNC-76 (Toda et al., 2008). Similarly, the LRRK2 kinase regulates autophagosome AT. Hyperactivation of LRRK2 phosphoactivity led to a specific decrease in AT of autophagosomes and impairment of autophagosomal maturation (Boecker et al., 2021), while inhibition of LRRK2 led to increased AT of alpha-synuclein (Brzozowski et al., 2021). Additionally, a role for VPS34 in mediating the attachment between ankyrin-B and the p62 subunit of dynactin for AT has been proposed. Knocking down VPS34 caused a reduction in VPS34 in neuronal processes and led to axonal swellings and disruption of AT of multiple organelles (Lorenzo et al., 2014). Thus, not only is autophagy tightly linked to AT, but autophagic kinases themselves regulate AT in specific ways.

### Regulation of axonal ER-phagy and mitophagy

Recent studies have provided key insights that both ER-phagy and mitophagy are highly regulated processes within axons. Inhibition of VPS34 led to accumulation of tubular ER in axons and activation of ER-phagy, indicating a role for VPS34 in regulating axonal ER-phagy (Kuijpers et al., 2021). Similarly, selective damage of mitochondria led to the recruitment of autophagosomes to damaged mitochondria within axons (Ashrafi et al., 2014). In a neuronal ischemia model, damaged axonal mitochondria had increased retrograde transport to the soma for mitophagy (Zheng et al., 2019). Indeed, mitophagy in neurons has been primarily located in the soma in *Drosophila* models (Devireddy et al., 2015; Sung et al., 2016) and in *in vitro* neuronal cultures (Evans and Holzbaur, 2020). It is likely that initial activation of mitophagy of damaged mitochondria occurs locally in the axon, after which damaged mitochondria are transported to the soma to complete mitophagy. As detailed above, the autophagic kinase TBK1 regulates mitophagy *via* phosphorylation of p62/SQSTM1 and Rab7A (Matsumoto et al., 2015; Heo et al., 2018). Thus, autophagic kinases specifically regulate axonal ER-phagy and mitophagy.

### Regulation of axonal degeneration and regeneration

Autophagic kinases play an essential role in regulation of axonal degeneration and regeneration. ULK1 negatively regulates axonal growth and regeneration, likely through activation of autophagy. In a siRNA forward genetic screen, knocking down ULK1 increased neurite outgrowth and enhanced neurite regeneration after transection (Loh et al., 2008). Additionally, axonal injury led to an upregulation of ULK1 as well as other autophagy proteins within injured axons (Ribas et al., 2015). In fact, expressing a dominant negative ULK1 in rats or treatment with a ULK1 inhibitor showed reduced autophagy and axonal degeneration in response to axotomy (Vahsen et al., 2020). Thus, ULK1 likely inhibits axonal outgrowth *via* activating autophagy to regulate turnover of membrane constituents.

On the other hand, other autophagic kinases promote axonal regeneration. The autophagic kinase VPS34 may positively regulate axonal function, as conditional knockout of VPS34 in mouse sensory neurons led to marked axonal degeneration in large-diameter axons (Zhou et al., 2010). The autophagic kinase mTOR has also been implicated in axonal regeneration. Activation of mTOR through inhibition of its upstream negative regulators PTEN or TSC1 enhanced axon regeneration in retinal ganglion cells (Park et al., 2008). Intriguingly, multiple mechanisms have been shown for mTOR regulation of axonal regeneration. First, in addition to regulating autophagy *via* inhibiting ULK1, mTORC1 also promotes local translation in response to axonal injury *via* phosphorylation of S6K and 4E-BP (Brunn et al., 1997; Laplante and Sabatini, 2012). In the peripheral nervous system, injured sensory axons locally upregulate mTOR (Abe et al., 2010), and inhibition of mTOR activity led to inhibition of local axon protein synthesis and reduced neuronal survival (Terenzio et al., 2018). This indicates that mTOR mRNA is present in the axon to rapidly upregulate local protein translation in response to axonal injury (Terenzio et al., 2018). Genetic knock down of mTOR and Raptor, components of mTORC1, suppressed axonal regeneration in dorsal root ganglion neurons (Chen et al., 2016). Raptor deletion reduced Stat3 signaling, a known regulator of axonal regeneration (Bareyre et al., 2011), indicating that another role for mTOR in promoting axonal regeneration may be through activation of Stat3 (Chen et al., 2016). Thus, autophagic kinases utilize distinct pathways to regulate axonal degeneration and regeneration.

### Regulation of the presynaptic terminal

Synapse formation and activity are also regulated by autophagic kinases. In *C. elegans*, the ULK1 ortholog UNC-51 is colocalized with its regulator ubiquitin ligase RPM-1 at axon termination sites (Crawley et al., 2019). Inhibition of UNC-51 by RPM-1 is required for axon termination and for maintenance of synapses through restriction of autophagosome formation in the distal axon (Crawley et al., 2019). Similarly in *Drosophila*, overexpression of the ULK1 ortholog *atg1* or treatment with rapamycin to inhibit *tor* (the *Drosophila* ortholog of mTOR) increased the number of neuromuscular junction boutons, which could be rescued with a null allele for the downstream autophagy gene *atg18*, signifying that motor neuron presynaptic terminals are regulated by autophagy (Shen and Ganetzky, 2009). In the central nervous system, synapses in dopaminergic neurons are also regulated by mTOR. Inhibiting mTOR with rapamycin led to an increase of axonal autophagosomes along with a decrease in synaptic vesicle number and dopamine transmission, suggesting that mTOR may negatively regulate synaptic transmission (Hernandez et al., 2012). These studies show opposing roles for mTOR and ULK1 in synapse regulation, and underscore the role of autophagy in regulating synapse homeostasis.

## Disrupted TBK1 activity in amyotrophic lateral sclerosis

Amyotrophic lateral sclerosis (ALS) is a progressive and fatal neurodegenerative disease. Pathologic hallmarks are the presence of cytoplasmic ubiquitinated aggregates, consistent with a defect in autophagy, and axonal degeneration of motor neurons. In support of a critical role for autophagy in ALS pathogenesis is the discovery of mutations in the autophagic kinase TBK1 as a cause of inherited ALS and frontotemporal dementia (Cirulli et al., 2015; Freischmidt et al., 2015; Williams et al., 2015). Postmortem neuropathologic findings showed p62/SQSTM1 and TDP-43 positive inclusions, indicating impaired autophagy. ALS-linked TBK1 mutations led to defective mitophagy, impaired autophagosome formation and impaired phagophore elongation (Moore and Holzbaur, 2016; Catanese et al., 2019; Harding et al., 2021). TBK1 has been linked to axonal dysfunction in several different ways. While TBK1 knockout or mutant G271R TBK1 mice did not have phenotypes alone, they exacerbated motor neuron (MN) denervation in SOD1^G93A^ mice (Brenner et al., 2019; Gerbino et al., 2020). Further evidence for the role of TBK1 affecting presynaptic terminals in ALS/FTD comes from a study in which overexpressing the TBK1 ortholog *ik2* in *Drosophila* rescued neuromuscular junction overgrowth in a model of FTD (Lu et al., 2020). Another link between TBK1 and endolysomal trafficking is that TBK1 directly phosphorylates Rab7a, a critical regulator of late endosomes. In fact, TBK1 loss of function in human iPS MNs and TBK1 patient-derived human MNs led to a reduction of Rab7a and deficient lysosomal activity (Hao et al., 2021). In axons, loss of TBK1 in human iPS MNs led to overactive spontaneous firing and impaired axonal regeneration, suggesting a link between impaired TBK1 regulation of endolysosomal trafficking and axonal dysfunction in ALS (Hao et al., 2021). These data indicate that ALS-causing TBK1 mutations may cause dysregulation of axonal function through multiple pathways, including autophagosome formation, mitophagy, and endolysosomal trafficking.

## Concluding remarks

Autophagic kinases play essential roles for autophagy in neurons. Additionally, autophagic kinases regulate diverse axonal functions including AT, synaptic maintenance and axonal degeneration and regeneration. ALS-causing mutations of the autophagic kinase TBK1 highlight the importance of these proteins in neurodegeneration. In fact, kinase activators and inhibitors are a growing class of therapeutics, making autophagic kinases appealing as treatment targets (Xiang et al., 2020). Pharmaceutical modulation of several of these kinases reviewed above are currently in development (https://clinicaltrials.gov, NCT04892017; https://clinicaltrials.gov, NCT02941523; Meunier et al., 2020). These examples highlight the feasibility of targeting autophagic kinases for therapeutic purposes. Further defining the precise mechanisms through which autophagic kinases regulate distinct axonal processes will aid the development of treatment targets for neurodegeneration.

## Author contributions

SB and DR wrote the manuscript. TL was responsible for the critical revision. All authors contributed to the article and approved the submitted version.
